# Severe vitamin D deficiency in 6 Canadian First Nation formula-fed infants

**DOI:** 10.3402/ijch.v72i0.20244

**Published:** 2013-04-16

**Authors:** Melissa L. Gross, Milton Tenenbein, Elizabeth A. C. Sellers

**Affiliations:** 1 Department of Paediatrics, Alberta Children's Hospital, Calgary, Canada; 2 Department of Paediatrics and Child Health, University of Manitoba, Winnipeg, Canada

**Keywords:** First Nation, infant, vitamin D, hypocalcaemia, seizures

## Abstract

**Background:**

Rickets was first described in the 17th century and vitamin D deficiency was recognized as the underlying cause in the early 1900s. Despite this long history, vitamin D deficiency remains a significant health concern. Currently, vitamin D supplementation is recommended in Canada for breast fed infants. There are no recommendations for supplementation in formula-fed infants.

**Objective:**

The objective of this report is to bring attention to the risk of severe vitamin D deficiency in high risk, formula fed infants.

**Design:**

A retrospective chart review was used to create this clinical case series.

**Results:**

Severe vitamin D deficiency was diagnosed in six formula-fed infants over a two-and-a-half year period. All six infants presented with seizures and they resided in First Nation communities located at latitude 54 in the province of Manitoba. While these infants had several risk factors for vitamin D deficiency, they were all receiving cow's milk based formula supplemented with 400 IU/L of vitamin D.

**Conclusion:**

This report suggests that current practice with regards to vitamin D supplementation may be inadequate, especially for high-risk infants. Health care professionals providing service to infants in a similar situation should be aware of this preventable condition. Hopefully this would contribute to its prevention, diagnosis and management.

Rickets was first described in the 17th century by Dr. Daniel Whistler and Professor Francis Glisson (1). However, it was not until the early 1900s that vitamin D deficiency was recognised as the underlying cause, and that the importance of sunlight to maintain adequate vitamin D levels was appreciated. Vitamin D fortification of food began in an attempt to address this widespread deficiency and ensure adequate intake. In Canada, the fortification of evaporated and dried milks began in 1950, while in 1965 the regulations were changed to include fluid milk (2). Infant formula is also fortified with 400 IU/L (3).

Despite the long history of recognition of this problem, vitamin D deficiency continues to remain a significant health issue (4–12). The incidence of vitamin D deficiency in Canada is not known. The Canadian Paediatric Surveillance Program (CPSP) recently included vitamin D deficiency rickets in its national surveillance program (13). One-hundred-and-four cases of vitamin D deficiency rickets were reported over a 2-year period in infants and toddlers, 96.2% of whom had been breast-fed. 86.5% of these infants had not received vitamin D supplementation. Three cases were formula-fed, 2 of which are included in this report along with 4 others.

From May 2003 to March 2005, we diagnosed vitamin D deficiency in 6 exclusively formula-fed First Nation infants presenting with hypocalcaemic seizures. Approval for submission of this report was received from the Human Research Ethics Board, Faculty of Medicine, University of Manitoba.

## Cases

The clinical information of the 6 cases is presented in Table I. Age at presentation varied from 5 days to 7 weeks. All 6 infants were of First Nation descent, full term, and had no significant medical history. All were from isolated First Nations communities >400 km northeast of Winnipeg (approximately the 54th parallel of latitude). They were all exclusively fed a vitamin D fortified cow's milk-based infant formula and were gaining weight appropriately. Proper formula preparation was verified. All 6 infants presented with seizures, either focal or generalised.

**Table I:** Clinical presentation and serum biochemistry profiles at presentation of 6 confirmed cases of severe vitamin D deficiency

Case	Age at diagnosis (day)	Presentation	Calcium (mmol/L); total/ionised	Phosphate (mmol/L)	ALP (IU/L)	PTH (ng/L)	25-Vitamin D (nmol/L)	Gender	Month of presentation	Maternal 25-vitamin D (nmol/L)
1	49	Focal seizures	1.54/0.97	2.23	675	266	<15	Male	March	N/A
2	6	Focal to generalised seizures	1.47/0.95	3.55	435	52	25	Female	January	16
3	5	Generalised seizures	1.35/na	3.29	310	16	16	Male	June	<15
4	42	Focal seizures	1.57/0.99*	3.2	632	73	<15	Male	November	N/A
5	42	Generalised seizures	1.55/na	1.87	586	175	<15	Female	January	N/A
6	35	Focal seizures	1.52/0.84	2.81	573	184	<15	Female	May	N/A

ALP=alkaline phosphatase, PTH=parathyroid hormone.

* Ionised calcium was drawn 2 hours after patient received bolus of IV calcium.

Normal values: Total calcium (2.10–2.60 mmol/L); ionised calcium (>1.0 mmol/L); phosphate (1.28–2.26 mmol/L); ALP (117–352 IU/L); PTH (7–50 ng/L), 25-vitamin D (75–200 nmol/L).

The profiles of the cases at presentation are summarised in Table I. The parathyroid hormone (PTH) level was elevated in all but the youngest infant. The 25-vitamin D level was low in all infants. The 25-vitamin D levels were available in the mothers of infant No. 2 and 3 and both were deficient (16 nmol/L and <15 nmol/L respectively; normal 75–200 nmol/L). Of the 2 mothers with recorded data, consumption of milk was minimal. None of the mothers were taking prenatal supplements.

Once therapy was initiated, none of the infants had recurrence of seizures. Cases 1, 2, 4, and 6 were initially treated with calcitriol, vitamin D3 supplements (2000 IU/day) (cholecalciferol), and oral calcium supplements. Calcitriol was discontinued after 2 weeks. Cases 3 and 5 were treated with cholecalciferol and calcium supplements in hospital and then discharged home on vitamin D 2000 IU/day. Vitamin D3 (2000 IU/day) was continued in all 6 infants for 2 months and then decreased to 400 IU/day.

## Discussion

Within a relatively short time period, we have seen 6 cases of severe vitamin D deficiency presenting with hypocalcaemic seizures in formula-fed First Nation infants less than 2 months of age. All had multiple risk factors for vitamin D deficiency including darker skin pigmentation, limited cutaneous sun exposure, residence at a northern latitude of 54°, and confirmed or suspected vitamin D deficiency in their mothers. All presented in the late autumn, winter or early spring when cutaneous synthesis of vitamin D would be minimal. In the 2 youngest infants in our series, it is likely that profound maternal vitamin D deficiency was the most significant factor leading to the early presentation of these infants.

The PTH level in the youngest infant in this series was not elevated in response to hypocalcaemia (PTH 16 ng/L). This may represent the physiologic blunting of PTH response in the newborn period (14). Case 1 had a family history of seizures, and may have had a lower seizure threshold, possibly explaining why this infant presented with seizures when the ionised calcium was 0.97 mmol/L.

The infants in this series were exclusively bottle fed a cow's milk-based formula fortified with 400 IU/L of vitamin D. They differ from the infants with vitamin D deficiency in the CPSP study, 96.2% of whom were breast fed (13). The infants in our report were also significantly younger than those from the CPSP study. In the CPSP study, 34 out of 104 children with vitamin D deficiency presented under 1 year of age. Of these infants, mean age at presentation was 0.6 years. It is likely that the younger age of presentation in our series reflects more profound deficiency and very likely severe maternal deficiency.

There have been several recent reports of severe vitamin D deficiency presenting with hypocalcaemic seizures in breast fed infants. In general, these infants were older at presentation when maternally derived stores would be expected to be depleted (15–19). Vitamin D deficiency presenting as hypocalcaemic seizures has also been reported in 3 infants fed soy-based infant formula (20). A series of infants (<13 months of age) presenting with hypocalcaemic seizures was previously reported by investigators at the Winnipeg Children's Hospital Health Sciences Centre in Manitoba almost 4 decades ago. These infants were presumed to be vitamin D deficient though vitamin D levels were not consistently available. This report does not detail the infant feeding practices (21).

The current statement put forth by the Canadian Paediatric Society, Dieticians of Canada, and Health Canada on nutrition for term infants recommends supplementing with 400 IU/day of vitamin D only for breast fed infants (22). There is no recommendation for supplementation in formula-fed babies. In an attempt to address the concerning frequency of vitamin D deficiency in Aboriginal infants, the Canadian Paediatric Society advocates for an increase to 800 IU/day in northern native communities during the winter months (23). The Canadian Paediatric Society statement does not clearly differentiate breast-fed vs. formula-fed infants but does suggest that infant formulas should be an adequate source of vitamin D as long as the infant consumes “adequate quantity” (23). It seems unlikely that infants in the first few months of life would drink 2 L of formula/day, the volume necessary to receive 800 IU/day of vitamin D. Four of our cases presented during the autumn and winter months, the other two infants presented during the early spring. None of these infants were receiving daily vitamin D supplementation. The American Academy of Paediatrics now recommends that formula-fed newborns who do not consume a minimum of 1 litre of formula per day receive supplementation to achieve an intake of 400 IU vitamin D/day (24). A recent telephone survey of mothers of full-term infants in Montreal identified that infants consuming mixed feedings (breast and formula) and those exclusively formula-fed were at high risk of not meeting recommended vitamin D intake in the first 6 months of life (25).

Transplacental transfer of vitamin D stores from the mother to foetus occur during the third trimester. Adequate transfer of stores depends on the vitamin D status of the mother. In infants born to a vitamin-D-replete mother, these stores are exhausted by approximately 8 weeks of age (26). Maternal vitamin D deficiency remains a common concern (27). In the mothers of the infants presented in this report milk consumption was minimal and none were taking prenatal vitamins. They were also all living at northern latitude and had darker skin pigment. These factors contribute to vitamin D deficiency in the mothers, and therefore, poor placental transfer to the infant (28). In addition, Aboriginal women from Manitoba have been shown to have lower vitamin D levels than Caucasian women even though vitamin D intake did not differ between the groups. In this study, >30% of Aboriginal women had vitamin D levels <37.5 nmol/L compared to 18.6% of Caucasian women (29). Vitamin D levels were deficient in both the mothers in this report who had levels drawn. Given the young age of the cases presented (mean age 30 days), maternal vitamin D deficiency is likely a major contributing factor in all the cases presented. The reason for poor use of prenatal vitamins in this population is not clear. While the mothers reside in remote First Nations communities, access to prenatal care is local and prenatal supplements are available at no cost to the individual.

Vitamin D deficiency continues to remain a significant health issue in Canadian northern communities. While our 6 infants had numerous risk factors for vitamin D deficiency, they were not breast fed, which was the most consistent finding in previous reports (13, 15–19). The infants we report presented with hypocalcaemic seizures and represent the most severe spectrum of the problem. It is likely that other asymptomatic infants with vitamin D deficiency remain undiagnosed from this high-risk population. This raises the question of whether 400 IU of vitamin D per litre of formula is sufficient. Policies have been put forth to address this issue in the high risk First Nation and Inuit populations; however, in our experience, they are either not being implemented and/or compliance remains a barrier. This underlies the importance of awareness among primary care providers.

## Conclusion

We suggest that the amount of vitamin D supplementation needed to prevent deficiency in infants residing in communities in the temperate region should be revisited, particularly for those at high risk for prenatal deficiency. Attention also needs to be focused on the prevention of maternal vitamin D deficiency. In addition, education and other strategies are needed to ensure that current and new policies are effectively implemented.
